# Optical turbulence and spectral condensate in long fibre lasers

**DOI:** 10.1098/rspa.2012.0037

**Published:** 2012-05-02

**Authors:** E. G. Turitsyna, Gregory Falkovich, Atalla El-Taher, Xuewen Shu, Paul Harper, Sergei K. Turitsyn

**Affiliations:** 1 Photonics Research Group, Aston University, Birmingham B4 7ET, UK; 2 Physics of Complex System, Weizmann Institute of Science, Rehovot 76100, Israel

**Keywords:** optical turbulence, Raman fibre lasers, nonlinear optics, spectral condensate

## Abstract

We study numerically optical turbulence using the particular example of a recently created, ultra-long fibre laser. For normal fibre dispersion, we observed an intermediate state with an extremely narrow spectrum (condensate), which experiences instability and a sharp transition to a fluctuating regime with a wider spectrum. We demonstrate that the number of modes has an impact on the condensate's lifetime. The smaller the number of modes, the more resistant is the condensate to perturbations. Experimental results show a good agreement with numerical simulations.

## 1. Introduction

Raman fibre lasers exploit the effect of simulated Raman scattering to shift the generated spectrum from pumping towards longer wavelengths. Raman fibre lasers are very attractive pump sources for distributed Raman amplification, which is one of the important enabling technologies in high-speed optical communication, as shown by Stolen & Ippen (1973), Mollenauer *et al.* (1986), Chernikov *et al.* (1999), Kurukitkoson *et al.* (2001) and Headley & Agrawal (2004). Using fibre Bragg gratings (FBGs) as cavity reflectors at the Stokes wavelength, it is possible to achieve lasing in a fibre waveguide with length of the order of several kilometres, as was first shown by Grubb *et al.* (1995). Recent developments in fibre lasers have shown new interesting applications such as quasi-lossless transmission using ultra-long fibre lasers (Ania-Castañón *et al.* 2006), distributed sensing (Frazao *et al.* 2009) and random fibre lasers (Turitsyn *et al.* 2010). It has also been shown recently (Babin *et al.* 2007*a*,*b*; Turitsyna *et al.* 2009; Randoux *et al.* 2011 and references therein) that fibre lasers present a unique test-bed for experimental studies of one-dimensional wave turbulence (Zakharov *et al.* 2001; Suret *et al.* 2010) that is, in turn, directly relevant to operation and performance of such practically important photonic devices. Various interesting examples of optical turbulence have been studied recently (Bortolozzo *et al.* 2009; Hammani *et al.* 2010; Klaers *et al.* 2010; Kibler *et al.* 2011). There has been a significant interest in optical wave turbulence in optical fibres in supercontinuum generation (Barviau *et al.* 2009*a*,*b*), with Raman effects (Pitois *et al.* 2006; Picozzi *et al.* 2008; Suret *et al.* 2010) and with reviews reported on condensation of classical nonlinear waves (Düring *et al.* 2009).

One of the most exciting subjects in modern turbulence studies is the appearance of turbulence and strong fluctuations in physical situations where the coherent (laminar) state is stable with respect to small perturbations. This is the case for turbulence occurring in a normal-dispersion fibre, which places it in the same class as the most ubiquitous turbulence, such as one in a pipe. In a pipe, the probabilistic nature of the transition to turbulence was recently revealed (Avila *et al.* 2011). Here, we find that the condensate destruction is also of probabilistic nature, thus placing fibre-optic turbulence into a wider context.

In this work, we also demonstrate the impact of optical wave turbulence on spectra and coherence of radiation generated in continuous wave fibre lasers with the Fabry–Perot resonator. For normal dispersion of the cavity, we investigated how the number of longitudinal modes involved affects the existence of condensate, and how resistant the condensate state is to perturbation. We hope that our work will contribute to general studies of wave turbulence (Zakharov *et al.* 1992; Lukaschuk *et al.* 2009; Nazarenko *et al.* 2010).

## 2. A laser system scheme and a basic mathematical model

**Figure 1.**
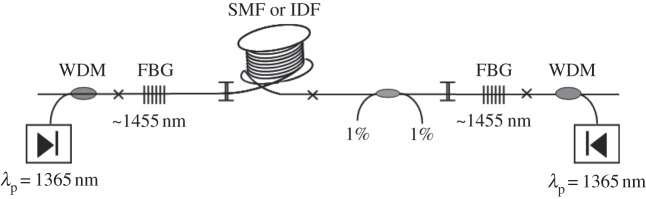
Laser system set-up. IDF, inverse dispersion fibre; SMF, single-mode fibre; WDM, wavelength-division multiplexing.

We consider the system described by Ania-Castañón *et al.* (2006), which is of importance for the so-called quasi-lossless signal transmission around 1555 nm in optical fibre spans supported by Raman amplification based on pumping sources operating at 1365 nm and FBGs at the first Stokes wavelength of 1455 nm (figure 1). This scheme provides rather uniform spatial distribution of generated Stokes wave power at 1455 nm along the cavity formed by FBGs.

For numerical simulations, we use the mathematical model, introduced in Babin *et al.* (2006, 2007*a*), that presents the standard round trip evolution equation for the longitudinal modes (*E*_*n*_) of the envelope, which can be derived from the generalized Schrödinger equations for backward and forward Stokes waves,
2.1
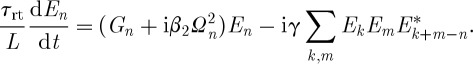
Here, *τ*_rt_=2*Ln*/*c* is the round trip time, *L* is the resonator length and *c* is the speed of light; the terms on the right-hand side describe, respectively, gain/loss (*G*_n_), group-velocity dispersion (*β*_2_*Ω*^2^_n_∝*n*) and the four-wave nonlinear interaction (including self-phase modulation) induced by the Kerr nonlinearity (*γ*). Equation (2.1) has the simplest stationary solution in the form of an ideal one-mode (or a pair of modes) condensate that corresponds to the maximum of *G*_n_. Recall that such a monochromatic wave is linearly unstable in the case of an anomalous dispersion (the so-called modulational instability) and is linearly stable in the case of a normal dispersion. It is thus no surprise that the laser radiation has a finite spectral width for an anomalous dispersion; however, it came as much of a surprise that the spectrum can be even wider for a normal dispersion, as discovered by Turitsyna *et al.* (2009). Appearance of wide spectra and strong fluctuations (optical turbulence) in a normal-dispersion fibre is the main subject of study in this work.

For long-cavity fibre lasers, the actual number of excited modes can be huge (up to 10^8^). For many interacting modes sharing between them a finite generated power, a natural first step is to assume the interaction as weak (weak-turbulence approach described by Zakharov *et al.* 1992), an effective nonlinearity/dispersion ratio of *ξ*=*γI*/|*β*_2_|*Ω*^2^_rms_≪1, the spectrum as wide and the phases of different modes as random. Prediction of the weak wave turbulence is that the results are insensitive to the sign of wave dispersion *β*_2_. Here, 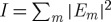 is the total generated power, 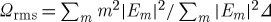 is the spectral bandwidth and *Δ* is the spectral separation between modes. However, as noted by Turitsyna *et al.* (2009) and will be shown later, changing the dispersion sign markedly changes the spectral shape and the statistics of the laser radiation. The detailed physical mechanisms behind such markedly different behaviour are not fully understood; however, similar to wave turbulence studies in hydrodynamics (Janssen 2003 and references therein), the main reason is the difference between linearly stable and unstable cases.

## 3. The impact of fibre dispersion

**Figure 2.**
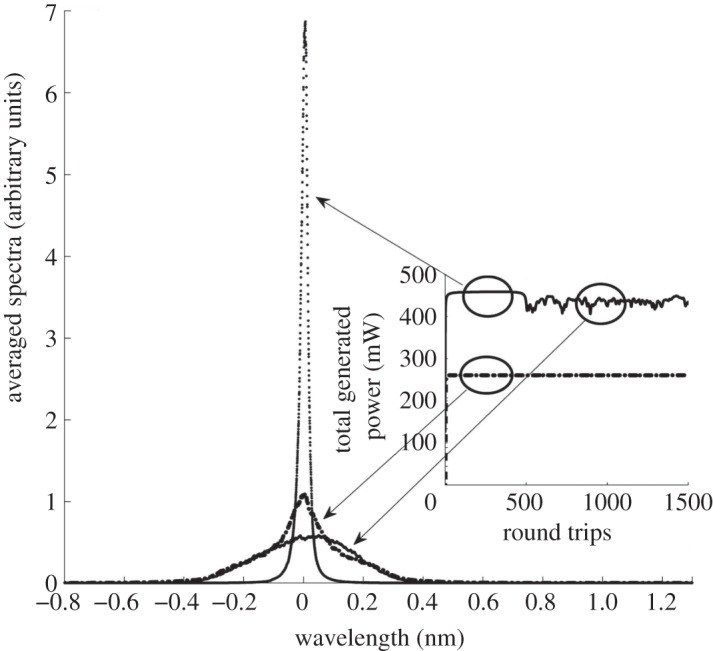
Spectra profiles and generated power evolution (inset picture) for different signs of dispersion and different numbers of round trips. Solid line, *β*_2_*L*=250 ps^2^; dotted line, *β*_2_*L*=250 ps^2^ and dashed-dotted line, *β*_2_*L*=250 ps^2^.

For numerical simulations, we have considered a 22 km fibre laser typical of many experiments performed at Aston University with a standard single-mode fibre (SMF) (Babin *et al.* 2006, 2007*a*,*b*, 2008). We treated different values of pump powers (from 400 to 1000 mW) and dispersion *β*_2_*L* in the interval (−300,300) ps^2^. We have discovered that in the case of anomalous dispersion (*β*_2_<0), the generated spectra became steady with only small fluctuations in the generated power (just a few mW) after only a few round-trip times. For normal dispersion (*β*_2_>0), a very narrow wave packet of a few modes, which we called condensate, is formed initially. It persists for a time depending on the number of modes and the absolute value of dispersion. During the condensate lifetime, the total intensity is constant with high accuracy (as seen in the inset of figure 2). The condensate destruction is manifested by a sharp transition to a wider spectrum and a lower mean power. That new (statistically steady) state is accompanied by stronger fluctuations, which seem to be a sign of bi-stability.

**Figure 3.**
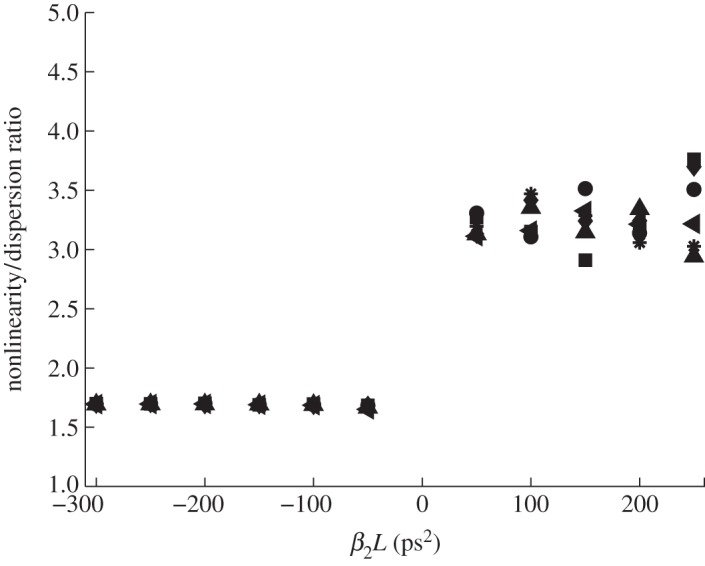
Nonlinearity/dispersion ratio for different pump powers and *β*_2_ values. Squares, pump power 500 mW; diamonds, pump power 600 mW; circles, pump power 700 mW; asterisks, pump power 800 mW; triangles, pump power 900 mW; inverted triangles, pump power 1000 mW.

Spectral broadening at *β*_2_>0 is due to the competition between four-wave mixing (FWM) and dispersion; so one can expect that the width is determined by the balance of dispersion and nonlinearity, *β*_2_|*Ω*^2^_rms_≅*γI*, i.e. comparable to that determined by modulation instability (MI). This would mean that the effective nonlinearity/dispersion ratio *ξ*=*γI*/|*β*_2_|*Ω*^2^_rms_ must stay approximately constant and of order unity. As seen in figure 3, *ξ* practically does not depend on |*β*_2_|*L* and the pump power. The effective dispersion is almost twice as large for *β*_2_<0 when it must balance both nonlinearities, MI and FWM, acting together to widen the spectrum. Because *ξ*>1, then in all these cases, most of the modes cannot be treated by the weak-turbulence approximation.

## 4. Experimental studies and comparison with numerical modelling

**Figure 4.**
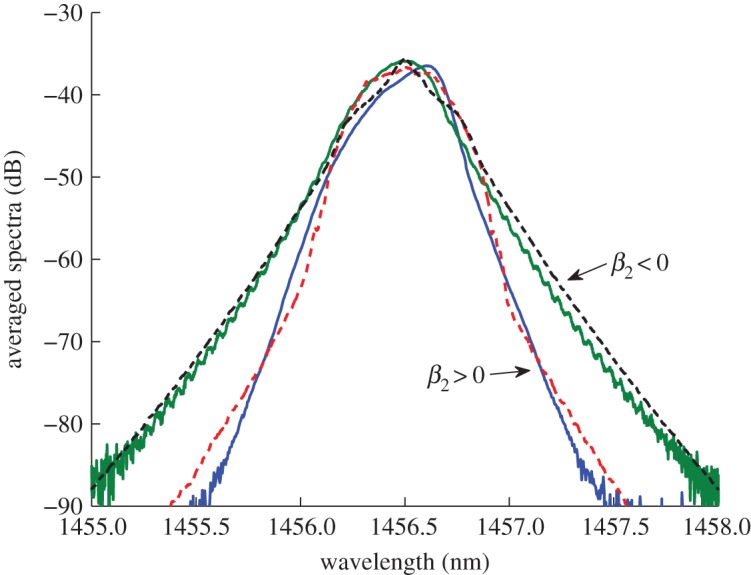
Comparison between numerical simulation and experiment for SMF and IDF fibres. Blue line, IDF experiment; green line, SMF experiment; red dashed line, IDF numerics; black dashed line, SMF numerics.

Experimental studies have been performed with two characteristic laser cavity lengths: 22 and 13 km. Experimental results for a 22 km SMF laser have been published previously (Babin *et al.* 2006, 2007*a*,*b*, 2008), and we used those experiments as a control case for an anomalous fibre dispersion. To clarify the difference between normal and anomalous (positive and negative) dispersions, we have performed control experiments with the same laser cavity length (13.5 km) built from two commercially available fibres: SMF with anomalous dispersion 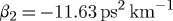 (here, *β*_2_*L*≈ =−157 ps^2^) and inverse dispersion fibre (IDF) with normal dispersion 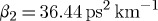 (here, *β*_2_*L*≈492 ps^2^). Numerical simulations with the number of modes less than 2^16^=65 536 demonstrated a rather long temporal existence of the condensate state. For a larger number of modes, the condensate state did exist, but did not last long and experienced transition to a strongly fluctuating regime. We have not yet observed the condensate state during the experiment, mainly because the number of modes is massively larger (not amenable for considering in numerical simulations). Figure 4 demonstrates good agreement between numerical and experimental results (here, the total pump power was 600 mW), which proves that our numerical model is quite accurate. It is seen from figure 4 that the wings of all spectra (as for normal and for anomalous dispersion) exhibit a linear slope on a log scale. This property was first reported for normal dispersion (Babin *et al.* 2007*a*). Here, we can observe the same for anomalous dispersion. This quite remarkable fact will be discussed and analysed in details elsewhere.

## 5. Spectral condensate in fibre lasers

### (a) Number of modes and condensate existence

Now we discuss the impact of a number of generated modes on the building of an equilibrium state. In a Fabry–Perot cavity, the spectral separation between laser modes is inversely proportional to the round-trip time *τ*_rt_,
5.1
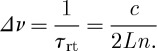
Here, *L* is the cavity length and *c*/*n* is the speed of light in the cavity. In the case of a fibre made of silica with some dopant, *n* is around 1.4–1.5 (we consider here *n*=1.45).

**Figure 5.**
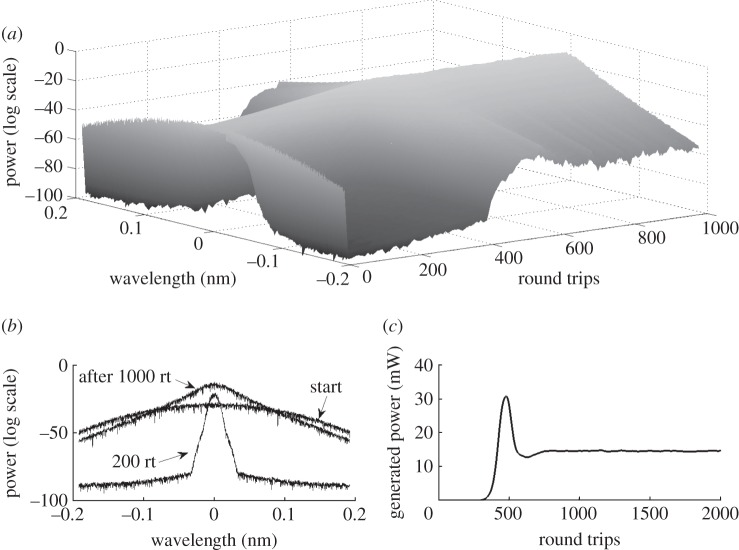
Fibre laser with a cavity of 1 km long IDF fibre (normal dispersion). (*a*) Spectra evolution for the first 1000 round trips (rt); (*b*) spectra profiles on different stages: at the start, after 200 round trips and after 1000 round trips; and (*c*) evolution of the generated power.

The number of modes generated in the laser can be estimated as *M*=*B*/*Δν*, where *B* is the laser linewidth that is related to some effects limiting the spectral interval over which the laser radiation is generated (e.g. FBGs are definitely involved, but their bandwidth should be used only as an estimate of the *B* parameter). One can estimate from (5.1) that for FBGs of 0.1 nm bandwidth (corresponding to *δ*_2_=277 nm^−2^) and 800 mW of total pump power, there will be approximately 32 280 modes, with the spectral separation between modes *Δ*=*λ*^2^/(2*Ln*)=7.3×10^−7^ nm. As we consider the spectral window eight times larger than the FBG bandwidth, it gives the total number of modes used in simulations 2^20^=1 048 576. Figure 5 demonstrates the evolution of the generated spectra for a fibre laser with a normal-dispersion cavity with this number of modes. As seen from figure 5, there is no clearly pronounced condensate state for this number of modes in the normal-dispersion cavity. Note, however, the appearance of a narrow spectrum at 200 round trips and a peak in total power at 500 round trips, as possible signs of an unrealized tendency to condensation.

**Figure 6.**
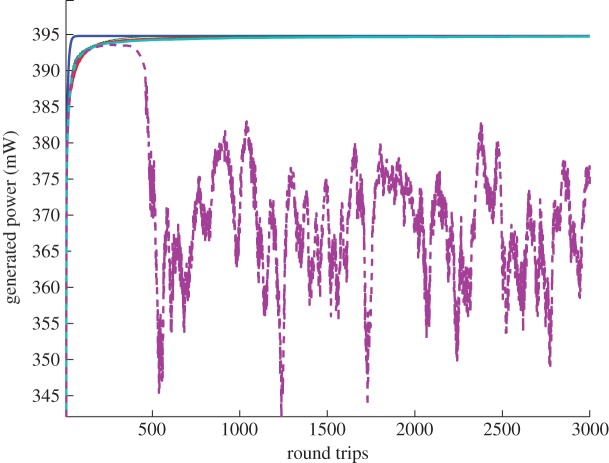
Generated power evolution for different numbers of modes for the IDF fibre (600 mW total pump power, 13.5 km cavity length). Dark blue line, 2^6^ modes; green line, 2^9^ modes; red line, 2^12^ modes; light blue line, 2^15^ modes; purple dashed line, 2^16^ modes.

**Figure 7.**
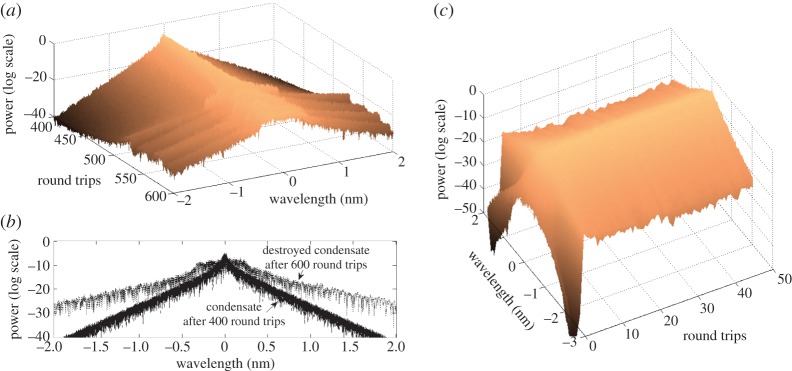
Spectra evolution for different types of fibre. (*a*) 13.5 km IDF fibre with 600 mW pump power. Condensate state exists for about 500 round trips, then is destroyed and starts oscillating. (*b*) The corresponding spectra after 400 round trips (solid line) and after 600 round trips (dashed line). (*c*) 13.5 km of SMF fibre. The generated spectra remain stable throughout the performance.

Figure 6 shows how evolution can be drastically changed by an increase in the total number of interacting modes. We observe that in the case of a normal-dispersion fibre (here, the IDF fibre) with a smaller number of modes, the generated power remains stable, with very little variations. In this case, the generated spectra are in the condensate state. With the increasing number of modes, the generated power evolution drops and starts oscillating. As a result, the spectra become very unstable, which can be seen in figure 7. Figure 7*a*,*b* shows the evolution of the spectra for a 13.5 km IDF fibre: transformation from the existing condensate to a destroyed one. As for the 13.5 km SMF fibre with anomalous dispersion, the generated spectra become stable after just a few round trips and do not change significantly with time (figure 7*c*). We have observed that distortion of the condensate state depends on the pump power, on initial phase conditions, but mostly on the number of modes.

It is interesting to investigate the behaviour of particular modes during the laser performance, both for normal and for anomalous dispersion fibres.

**Figure 8.**
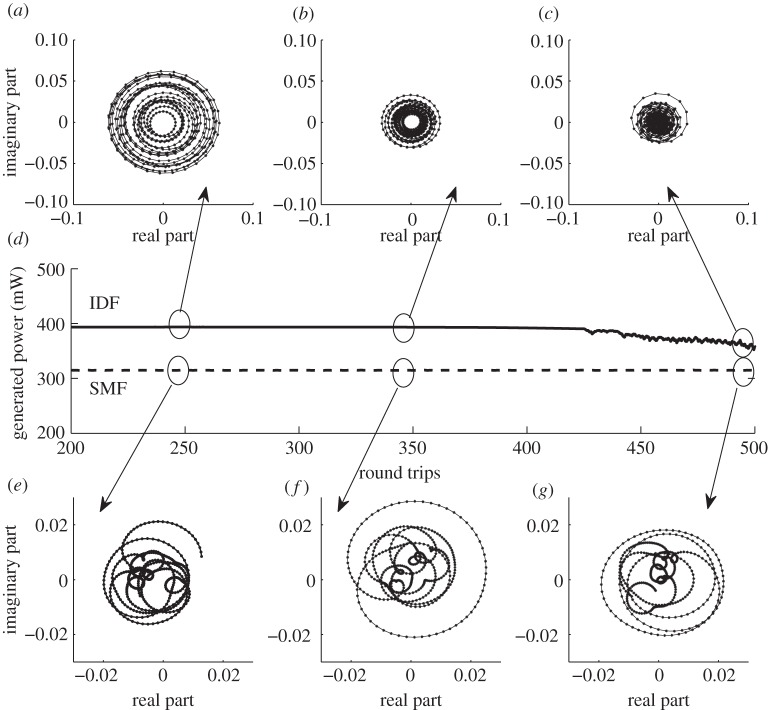
Plotted central mode behaviour (real part versus imaginary part) after different numbers of round trips. (*a*) IDf fibre from 245 to 250 round trips; (*b*) IDF from 345 to 350 round trips; (*c*) IDF from 495 to 500 round trips; (*e*) SMF from 245 to 250 round trips; (*f*) SMF from 345 to 350 round trips; (*g*) SMF from 495 to 500 round trips. (*d*) The generated power curve evolution: solid line, IDF fibre; dotted line, SMF fibre.

Figure 8*a*–*c* shows what happens to the central mode during a short life of condensate for an IDF fibre (13.5 km, 600 mW pump power, 2^16^=65 536 modes considered). It is seen how quasi-cyclical behaviour of the mode (figure 8*a*–*c*) changes to a chaotic one and how its amplitude shrinks (figure 8*c*). After about 500 round trips, the intensity, which was concentrated earlier in a few central modes, is now distributed more evenly between other modes. On the contrary, we observed a different evolution for the central mode for an SMF fibre (figure 8*e*–*g*). The amplitude of the central mode remains at about the same level throughout the propagation. We do not observe quasi-cyclical movements, as in the case of the IDF fibre. Here, we considered the same fibre length (13.5 km), 600 mW pump power and 65 536 modes. The (chaotic) trajectory in figure 8*c* fills the whole domain, in distinction from all other cases. It is also important to note the difference in the amplitude values of the central mode for both types of fibres. Although the generated power level is comparable (figure 8*d*), for the IDF fibre, the central mode amplitude is about four times larger than for the SMF fibre. It confirms that during the condensate state, most of the energy is concentrated in a few central modes, but after the condensate is destroyed, it becomes more evenly distributed (figure 8*c*).

### (b) Condensate resistance to small perturbations

**Figure 9.**
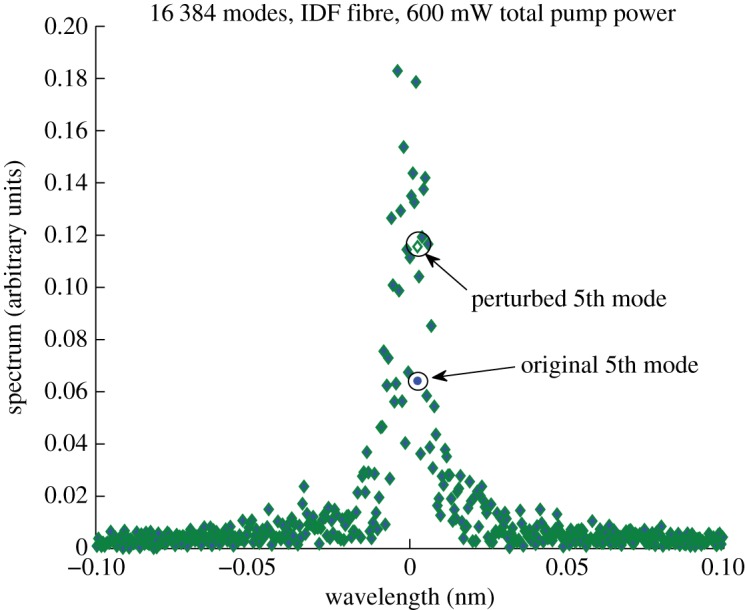
Spectrum used for adding some perturbation to the fifth from the centre mode. Blue circles, original field; green diamonds, with perturbation.

After establishing some conditions under which the condensate exists, we investigate how resistant it is to small structural perturbations. We consider the same IDF fibre with normal dispersion and the number of modes 2^14^. As shown earlier (figure 6), with this number of modes, the condensate ‘lives’ as long as the computing time permits. We extracted the field after the initial stage when the spectra became stable in the condensate state (after 1000 round trips), and then imposed a small perturbation (approx. 10%) to the mode fifth from the centre (figure 9).

**Figure 10.**
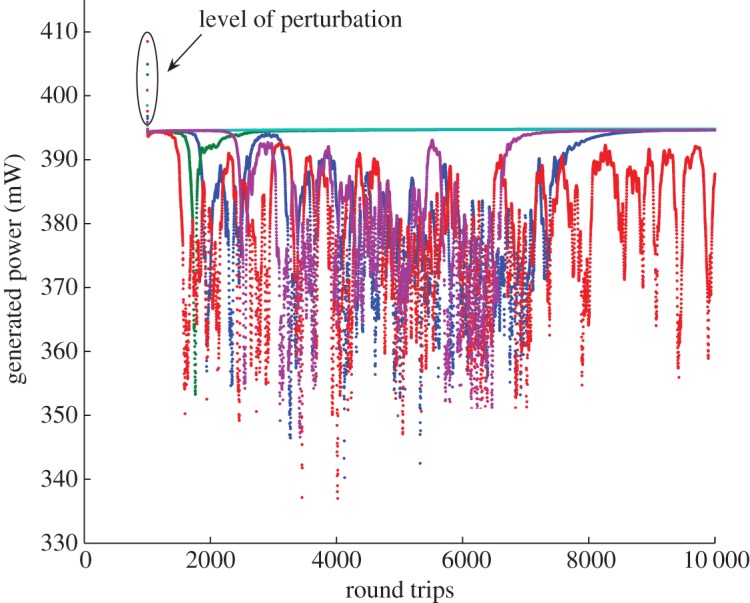
Different types of intensity evolution depending on the level of perturbation. Dark blue dots, early drop, late recover; green dots, early drop, early recover; red dots, early drop, not recovered; light blue dots, non-drop; purple dots, late drop, late recover.

**Figure 11.**
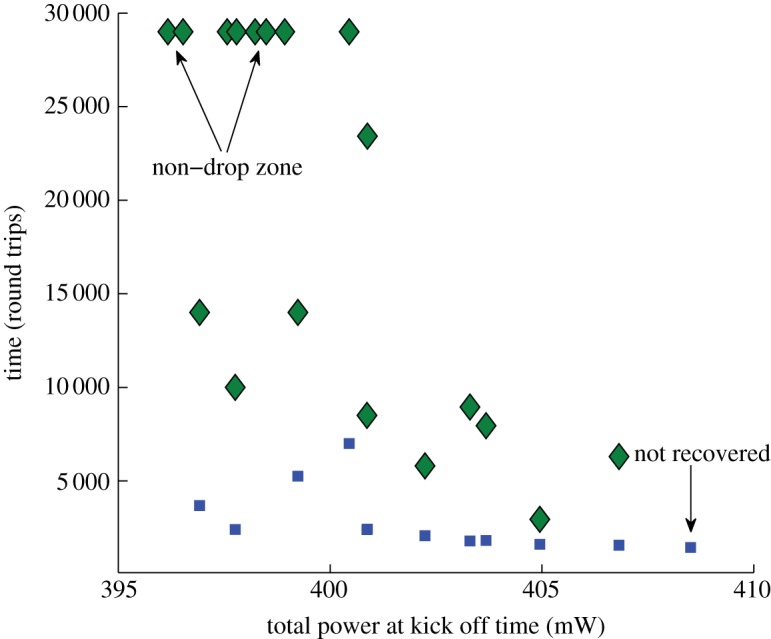
Dependence of condensate's drop and recovery time on the total generated power at the time of perturbation. Blue squares, drop time; green diamonds, recovery time.

The spectra evolution that followed and the intensity of the perturbed condensate state are presented in figures 10 and 11.

Depending on the level of perturbation, we observed different evolution of the generated intensity of the field: early drop and late recovery (blue dots), when the generated power dropped after the applied perturbation and has spent around 7000 round trips oscillating, but then ‘climbed back’ to its original level and remained there for the time of the numerical simulations, as shown in figure 10. We also observed the evolution of an early drop and early recovery (green line), late drop and late recovery (purple dots), non-drop (turquoise dots) and drop without recovery (red dots).

Figure 11 quantifies the dynamics shown in figure 10 by presenting dependence of drop time and recovery time for condensate for the IDF fibre, with total power of 600 mW, 16 384 modes, with perturbed fifth mode. The generated power of the condensate state is 394.5 mW and the drop time is measured at 393 mW.

In the case of the number of modes larger than 2^16^, the condensate state can also be observed. However, it did not last long and experienced sharp transition to a fluctuating regime. Experimental observation of the condensate is a challenging problem, and these studies will be presented in detail elsewhere.

## 6. Concluding remarks

We have demonstrated that the sign of cavity dispersion has a marked impact on optical turbulence that determines spectral and temporal properties of the generated radiation, directly related to the performance of an ultra-long fibre laser. For normal dispersion, we observe an intermediate state with an extremely narrow spectrum (condensate) that experiences instability and a sharp transition to a more fluctuating regime. The existence of this kind of state depends on the number of longitudinal modes. For a smaller number of modes, the condensate is more resistant to small perturbations. For anomalous dispersion, we have observed triangular spectra and more coherent temporal behaviour of generated radiation. The experimental results are in good agreement with numerical simulations. Our work shows the link between the very practical field of fibre lasers and the field of wave turbulence (Zakharov *et al.* 2001; Shrira & Geoigjaev 2010), offering new applications for both fibre lasers and turbulence theory.

## Appendix A

Here, we present the list of terms used in (2.1), which describes the mathematical model that we use in numerical simulations. *G*_n_=*g*−*δ*_n_/*L* describes the effective pumping *g* and FBG losses *δ*_n_. *L* is the resonator length, . Here, *α* (km^−1^) is distributed loss,  is the Raman gain coefficient in the optical fibre, and the averaged pump power  can be approximately expressed through the generated Stokes power *P* (see Babin *et al.* 2003 for details) as

A1

Here, *P*_p_(0) is the input (as regards the Raman fibre laser) pump power, *P* is the generated Stokes wave power averaged over time and the fibre cavity, *α*_p_ is the pump wave optical losses in the fibre, *λ*_p_=1365 nm and *λ*=1455 nm are the pump and Stokes wavelengths, respectively, and *δ*_n_ describes the combined effect of all lumped losses and frequency-dependent FBG losses. We use *δ*_n_=0.3+*δ*_2_(*nΔ*)^2^, corresponding to a Gaussian spectral response of the FBGs with *δ*_2_=3 nm^−2^. We put *Ω*_*n*_=*nΔ*, where *Δ*=*Ω*/*M* is a spectral separation between modes, *Ω* is the total spectral interval and *M* is the total number of modes used in numerical modelling. The window of *Λ*=*λ*^2^*Ω*/(2*πc*) (nm) significantly exceeded the spectral width in all cases. The group-velocity dispersion coefficient is *β*_2_ (ps^2^ km^−1^) and *Ω*_*n*_∝*n*.
